# Toward Therapeutic Drug Monitoring of Lenalidomide in Hematological Malignancy? Results of an Observational Study of the Exposure-Safety Relationship

**DOI:** 10.3389/fphar.2022.931495

**Published:** 2022-06-23

**Authors:** Zaiwei Song, Lan Ma, Li Bao, Yi Ma, Ping Yang, Dan Jiang, Aijun Liu, Lu Zhang, Yan Li, Yinchu Cheng, Fei Dong, Rongsheng Zhao, Hongmei Jing

**Affiliations:** ^1^ Department of Pharmacy, Peking University Third Hospital, Beijing, China; ^2^ Therapeutic Drug Monitoring and Clinical Toxicology Center, Peking University, Beijing, China; ^3^ Department of Hematology, Peking University Third Hospital, Beijing, China; ^4^ Department of Hematology, Beijing Jishuitan Hospital, Beijing, China; ^5^ Department of Hematology, Beijing Chaoyang Hospital, Capital Medical University, Beijing, China; ^6^ Department of Hematology, State Key Laboratory of Complex Severe and Rare Diseases, Peking Union Medical College Hospital, Chinese Academy of Medical Sciences and Peking Union Medical College, Beijing, China

**Keywords:** lenalidomide, hematological toxicity, therapeutic drug monitoring, trough concentration, hematological malignancies

## Abstract

**Objective:** Continuous lenalidomide (LEN) therapy is important to achieve a therapeutic effect in patients with multiple myeloma (MM) and non-Hodgkin lymphoma (NHL). However, despite dose adjustment according to kidney function, many patients discontinue LEN therapy because of hematological toxicity. To date, therapeutic drug monitoring (TDM) of LEN has not been performed in oncology, and no target concentration level has been yet defined. The aim of this study was to evaluate the exposure-safety relationship of LEN and determine the target concentration for toxicity.

**Materials and Methods:** A prospective observational study was designed and implemented. Blood samples were collected at 0.5 h (trough concentration, C_min_) before oral administration and 1 h (C_1h_) thereafter on the day. Clinical data were gathered from patients’ medical records and laboratory reports. Outcome measures of hematological toxicity were defined by the Common Terminology Criteria for Adverse Events. The concentration values were dichotomized by receiver operating characteristic (ROC) curve analysis, and the association between exposure and outcome was determined using the logistic regression model.

**Results:** Out of the 61 patients enrolled in this study, 40 (65.57%) had MM, and 21 (34.43%) had NHL. Hematological toxicity was reported in 15 (24.59%) patients. The LEN C_min_ showed remarkable differences (*p =* 0.031) among patients with or without hematological toxicity, while no association between C_1h_ values and toxicity was noted (*p*>0.05). By ROC analysis, a C_min_ threshold of 10.95 ng/mL was associated with the best sensitivity/specificity for toxicity events (AUC = 0.687; sensitivity = 0.40; specificity = 0.935). By multivariate logistic regression, an LEN C_min_ below 10.95 ng/mL was associated with a markedly decreased risk of hematological toxicity (<10.95 ng/mL vs. >10.95 ng/mL: OR = 0.023, 95% CI = 0.002–0.269; *p* = 0.003).

**Conclusions:** We demonstrate that the LEN trough concentration correlates with hematological toxicity, and the C_min_ threshold for hematological toxicity (10.95 ng/mL) is proposed. Altogether, LEN TDM appears to be a new approach to improve medication safety and achieve continuous treatment for patients with NHL or MM in routine clinical care.

## Introduction

Non-Hodgkin’s lymphoma (NHL) is a cancer that develops in white blood cells called lymphocytes, representing the most frequent hematological malignancy. It is estimated that NHL was responsible for 544,000 new cases and 260,000 deaths worldwide in 2020 (Sung et al., 2021). Multiple myeloma (MM), the second most common hematologic malignancy, is a plasma cell malignancy in which monoclonal plasma cells proliferate in bone marrow (Mitsiades et al., 2004). Currently, projections suggest that, as the two most common hematologic malignancies, the incidence of both NHL and MM will continue to increase (Cai et al., 2021; Zhou et al., 2021).

The past years have witnessed a dramatic shift in the treatment of NHL and MM, from chemotherapy to chemoimmunotherapeutic regimens, and now biological and targeted strategies. One such treatment option is lenalidomide (LEN), which is a thalidomide derivative known as an immunomodulatory drug (List et al., 2005). LEN’s significant activity in hematological malignancy has led to its incorporation into multiple treatment regimens (Gribben et al., 2015), such as the LEN plus rituximab (R^2^) regimen for NHL (Leonard et al., 2019), as well as regimens based on LEN plus dexamethasone for MM (Mateos et al., 2013). As an oral targeted antineoplastic agent, long-term and continuous LEN treatment is important to achieve a therapeutic effect (Kado et al., 2020).

However, a close link has been established between LEN therapy and severe hematological toxicities, including neutropenia, thrombocytopenia, anemia, and leukopenia. Almost half of the patients experienced hematological toxicity of any grade across studies, and the incidence of high-grade hematologic toxicity might be 30% or higher (Cheson et al., 2020). During real clinical practice, despite dose adjustments according to baseline kidney function, unacceptable hematological toxicity is still the most common factor preventing continuous therapy with LEN. In addition to possibly causing treatment interruption, LEN-related hematological toxicity can affect patient adherence to treatment, increase the relapse risk and increase healthcare costs.

Therapeutic drug monitoring (TDM) is the clinical practice of measuring drug exposure at designated intervals to tailor drug doses, thereby optimizing outcomes in individual patients. The process of TDM is predicated on the assumption that there is a definable relationship between concentration and therapeutic or adverse effects (Kang and Lee, 2009). In terms of LEN, dose-limiting hematological toxicity has been observed, and dose modification or treatment interruption according to the severity of myelosuppression is recommended (Ludwig et al., 2018). Furthermore, a high area under the plasma concentration-time curve (AUC) of LEN has been shown to result in severe adverse events (Chen et al., 2013; Kobayashi et al., 2018). However, accurate measurement of the AUC requires collecting and analyzing multiple blood samples, which is both costly and time consuming for patients and clinical staff. Consequently, AUC-based TDM could be difficult to implement in clinical practice. There still exists a gap in the optimal index for TDM of hematological toxicity that can be used in clinical settings.

Herein, in the present study, we aimed to investigate the association between LEN exposure and its hematological toxicity and to determine concentration targets, which could be used in the TDM of LEN in patients with NHL or MM.

## Materials and Methods

### Patients

This was a prospective observational study that was in compliance with the Declaration of Helsinki and approved by the hospital medical science research ethics committee (No. M2021573). Patients provided written informed consent prior to enrollment. Adult patients with MM and NHL taking LEN capsules (Revlimid^®^, Celgene Corporation) for at least 3 days (steady state) and performing LEN concentration measurements during therapy between October 2021 and February 2022 were enrolled in this study. Patients taking any dose of LEN, pretreated with or without LEN, were eligible.

Patients were excluded if they had incomplete data, making it unable to assess whether the outcome event occurred; the clinical diagnosis was unclear; they had impaired kidney function with creatinine clearance (CCr) < 45 mL/min; they had impaired liver function with alanine aminotransferase (ALT) or aspartate aminotransferase (AST) greater than 5 times the upper limits of normal (ULN); they failed to take medicine as prescribed; they did not perform blood sample collection according to the prescribed time; and the plasma concentration of LEN was below the detection limit of 1 ng/mL.

### Data Collection

Data were gathered from patients’ medical records and laboratory reports, which included patients’ demographics, clinical data on the diagnosis (subtype and stage of MM or NHL), history of previous chemotherapy, details of LEN therapy (including number of courses, duration days, dosage, antineoplastic agents combined), other concomitant drugs, and biological results. The biological data of complete blood count (CBC, including white cells, neutrophils, platelets, hemoglobin) were tested and collected on the day blood plasma concentration was measured, whereas the baseline CBC was collected before taking LEN of this cycle. In addition, other laboratory test data, including serum creatinine (SCr), total protein, albumin, alanine aminotransferase (ALT), aspartate aminotransferase (AST), and alkaline phosphatase (ALP) values, were also collected. The creatinine clearance (CCr) was estimated using the Cockcroft-Gault formula.

### Measurement of Lenalidomide Concentration

Given that the half-life of LEN is approximately 3–5 h (Chen et al., 2017), LEN was considered to have achieved a steady-state plasma concentration after 3 days. Only patients who achieved steady state were included in the analysis; thus, we accepted that the blood samples could be collected on day 3 after starting the LEN therapy. Blood samples were collected at 0.5 h (trough concentration, C_min_) before oral administration and 1 h (C_1h_) thereafter on the day. The two blood samples need to be collected before meals in the morning to avoid the influence of food on drug absorption and plasma concentration, although LEN was administered without regard to food intake in daily clinical practice. Blood samples were drawn into EDTA tubes.

The plasma was stored at −80°C until analysis. LEN concentrations were measured using a validated high-performance liquid chromatography tandem-mass spectrometry method (HPLC–MS/MS). LEN-^13^C_5_ was used as the internal standard. The analyte was separated on a Waters Atlantis^®^ HILIC silica column (5 μm, 2.1 mm × 150 mm). The selected reaction monitoring transitions were 260.1→149.1 for LEN and 265.1→149.1 for the internal standard. The lower limit of quantification is 1 ng/mL for LEN. This method was developed and validated according to regulatory requirements. The inter-run precision and accuracy were less than 11.8% and 5.0%, respectively.

### Outcome Definition

Attending physicians and pharmacists evaluated and graded the hematological toxicity according to the Common Terminology Criteria for Adverse Events Version 5.0 (NCI-CTCAE), and the highest grade of all decreased blood cells was defined as the grade of severity of hematological toxicity in this study. The clinical outcome was classified into two categories: a group with hematological toxicity and a group with no hematological toxicity.

In patients with normal baseline counts, hematological toxicity was defined as grade 2 or higher hematological adverse events, including leukopenia (decreased white blood cell count), neutropenia (decreased neutrophil count), thrombocytopenia (decreased platelet count) and anemia (decreased hemoglobin count). In patients with abnormal baseline counts, hematological toxicity was defined as a count <75% of baseline and grade 2 or higher hematological adverse events.

### Statistical Analysis

The statistical analyses were performed with SPSS software version 27.0 (IBM Corp., Armonk, NY, United States). For quantitative data following a normal distribution, we calculated the mean with standard deviations (mean ± SD) and used a T test to determine the difference between the groups. For nonnormally distributed data, we calculated the median with interquartile range [median (IQR)] and used the Mann–Whitney U test to compare the difference between the groups. Categorical variables were expressed as frequencies and proportions (%), and the chi-squared test was used to compare the differences between the groups.

If a statistically significant difference in LEN concentrations was observed between the groups, receiver operating characteristic (ROC) curves were constructed to determine a concentration threshold associated with hematological toxicity. The best threshold was chosen using the Youden index, identifying the target concentration that might result in hematological toxicity. Patients’ exposure to LEN was dichotomized depending on this threshold.

To account for various potential risk factors for developing hematological toxicity and to reduce other potential bias, the associations between concentrations and hematological toxicity were further adjusted by the logistic regression model. First, univariate analysis was performed to identify possible factors. Variables with statistical significance (defined as *p* <0.05) in the univariate analysis, as well as those determined by reading relevant literature and combining clinical experience, including gender, age, weight, CCr, albumin, diagnosis (NHL or MM), and co-administration of cytotoxic antitumor drugs, were then included in the multivariate logistic regression using the Enter method. All statistical analyses were two-tailed, and *p* <0.05 was considered statistically significant.

## Results

### Patient Characteristics

**FIGURE 1:**
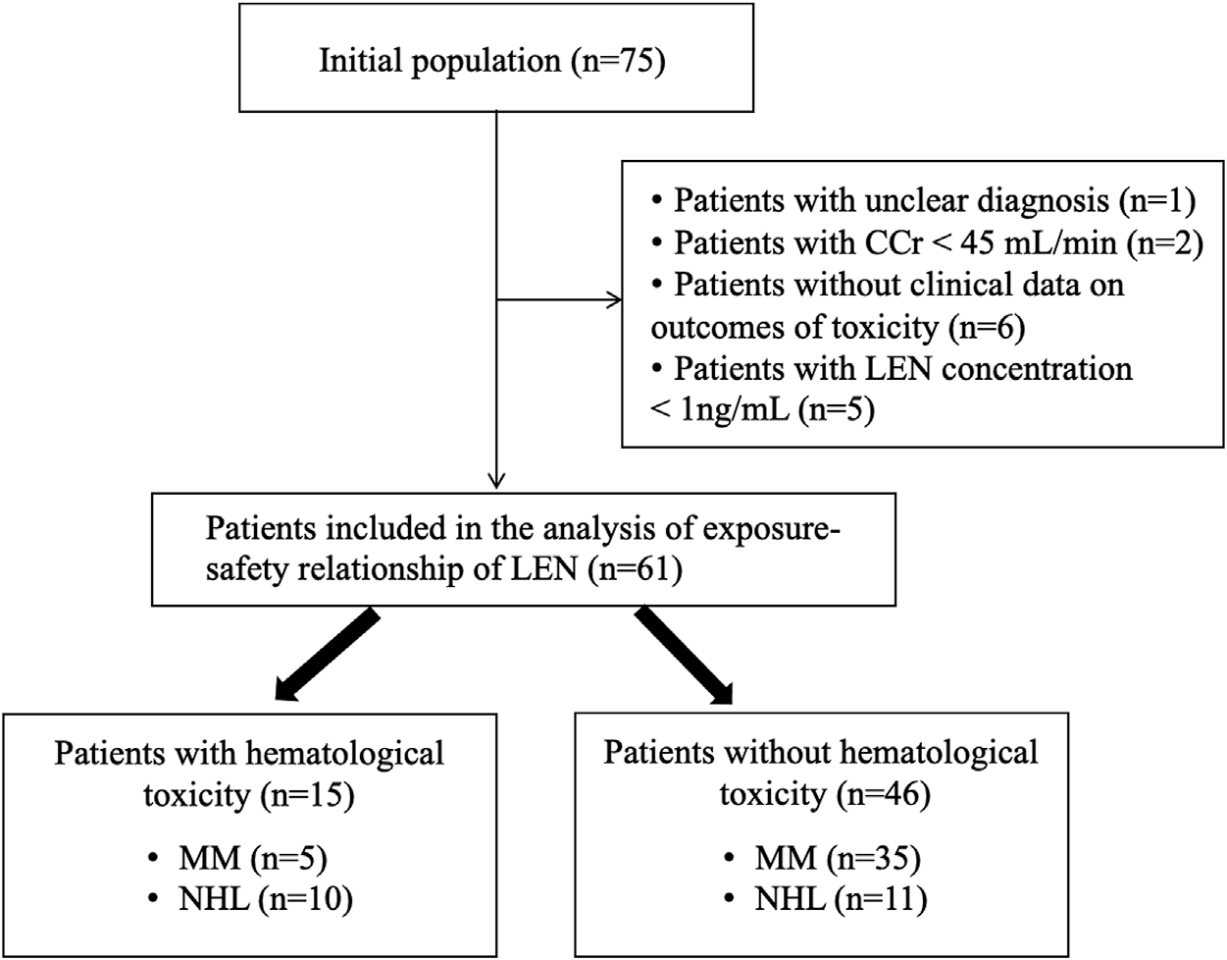
Flow chart of the study.

A total of 75 patients were screened initially, and only 61 (34 male, 55.74%) were included in the study. The other 14 patients were excluded as a result of unclear diagnosis, impaired kidney function with CCr less than 45 mL/min, missing clinical data, or LEN concentration below the detection limit of 1 ng/mL (Figure 1).

The demographic and clinical characteristics of the included patients are listed in Table 1. Out of the 61 patients enrolled in this study, hematological toxicity was observed in 15 (24.59%) patients. Patient demographics of the no-hematology toxicity group and hematology toxicity group were similar. Regarding the clinical diagnosis, 40 (65.57%) patients had MM, of which 26 were newly diagnosed; 21 (34.43%) patients had NHL, of which 5 were newly diagnosed. The median number of previous courses of immunochemotherapy was 0 (IQR, 0–5). Among 40 MM patients, the most common type was IgG (*n* = 19), followed by light chain (*n* = 15), IgA (*n* = 5), and IgD (*n* = 1). The most common Durie-Salmon (DS) stage was stage III (*n* = 33), followed by stage II (*n* = 5) and stage I (*n* = 2). Regarding the International Staging System (ISS), 23 patients had stage I disease, followed by stage III (*n* = 9) and stage I (*n* = 8) disease. Among 21 NHL patients, most had diffuse large B-cell lymphoma (DLBCL) (*n* = 11) and follicular lymphoma (FL) (*n* = 9), and only one had high-grade B-cell lymphoma (HGBL). According to the Ann Arbor Staging Classification, 19 and 2 patients had stage IV and stage II disease, respectively, of which almost half (*n* = 10) had B symptoms.

**TABLE 1:** Baseline demographic and clinical characteristics of the included patients.

Characteristic	No-Hematology toxicity group (n = 46) n (%)	Hematology toxicity group (n = 15) n (%)	Total (n = 61) n (%)
Gender			
Female	24 (52.17%)	10 (66.67%)	34 (55.74%)
Male	22 (47.83%)	5 (33.33%)	27 (44.26%)
Median age (IQR), years	62 (56–67)	59 (53–68)	62 (56–68)
Median weight (IQR), kg	64 (58–70)	65 (59–74)	64 (58–70)
Median BMI (IQR), kg/m^2^	23.50 (21.49–25.07)	23.80 (22.35–26.67)	23.51 (22.04–25.51)
Median BSA (IQR), m^2^	1.73 (1.59–1.83)	1.77 (1.62–1.87)	1.74 (1.60–1.84)
Diagnosis			
Multiple myeloma (MM)	35 (76.09%)	5 (33.33%)	40 (65.57%)
Non-Hodgkin lymphoma (NHL)	11 (23.91%)	10 (66.67%)	21 (34.43%)
Median SCr (IQR), μmol/L	61.5 (52.0–69.8)	61.0 (50.5–69.5)	61.0 (52.0–70.0)
Median CCr (IQR), mL/min	97.8 (74.5–109.3)	94.2 (72.7–102.7)	96.3 (74.5–108.5)
Median total protein (IQR), g/L	65.4 (60.2–69.6)	62.0 (59.3–73.1)	65.0 (59.8–70.0)
Median albumin (IQR), g/L	37.9 (35.7–41.0)	42.0 (36.8–43.1)	38.2 (35.7–41.9)
Median ALT (IQR), U/L	22.8 (13.3–27.2)	15.0 (10.0–35.5)	20.0 (11.1–30.0)
Median AST (IQR), U/L	22.0 (16.6–25.8)	17.0 (14.5–24.5)	21.0 (15.0–25.0)
Median ALP (IQR), U/L	79.0 (61.6–94.5)	69.0 (62.0–78.5)	76.5 (61.8–91.0)
Median courses of Lenalidomide (IQR), n	1 (1–3)	1 (1–3)	1 (1–3)
Median Lenalidomide duration (IQR), days	5 (5–7)	5 (5–6)	5 (5–6)
Dosage of Lenalidomide			
10 mg QD	5 (10.87%)	4 (26.67%)	9 (14.75%)
12.5 mg QD	4 (8.70%)	0	4 (6.56%)
25 mg QD	33 (71.74%)	11 (73.33%)	44 (72.13%)
25 mg QOD	4 (8.70%)	0	4 (6.56%)
Antineoplastic agents combined			
Targeted therapy^a^	41 (89.13%)	10 (66.67%)	51 (83.61%)
Glucocorticoids	36 (78.26%)	8 (53.33%)	44 (72.13%)
Cytotoxic antitumor drugs	4 (8.70)	5 (33.33%)	9 (14.75%)
Other concomitant drugs			
Aspirin	21 (45.65%)	9 (60.00%)	30 (49.18%)
Antiviral agents	19 (41.30%)	4 (26.67%)	23 (37.70%)
Antibacterial agents	7 (15.22%)	4 (26.67%)	11 (18.03%)
PPI or H2RA	5 (10.87%)	5 (33.33%)	10 (16.39%)

Abbreviations; IQR, Interquartile range; BMI,Body mass index; BSA, Body surface area; SCr, Serum creatinine; CCr, Creatinine clearance; ALT, Alanine aminotransferase; AST, Aspartate aminotransferase; ALP, Alkaline phosphatase; QD, Once a day; QOD, Every other day; PPI, Proton pump inhibitor; H2RA, H2 receptor antagonist.

a Targeted therapy includes bortezomib, isazomi, ibrutinib, zanubrutinib, orelabrutinib, rituximab and obinutuzumab.

The median number of LEN treatment courses was 1 (range 1–15), and the median days of LEN duration in this current cycle was 5 (range 3–21). The LEN dosage was classified into four categories: 10 mg QD, 12.5 mg QD, 25 mg QD, and 25 mg QOD. In terms of antineoplastic agents combined, LEN was combined with other targeted therapies (e.g., bortezomib, ibrutinib, rituximab) and glucocorticoids in most patients, and LEN monotherapy was administered in only 7 patients. In addition, patients were concomitant with other medications, including aspirin, antiviral agents, antibacterial agents, proton pump inhibitors (PPI) or H2 receptor antagonists (H2RA) and other drugs.

### Comparison of Plasma Concentrations

**FIGURE 2:**
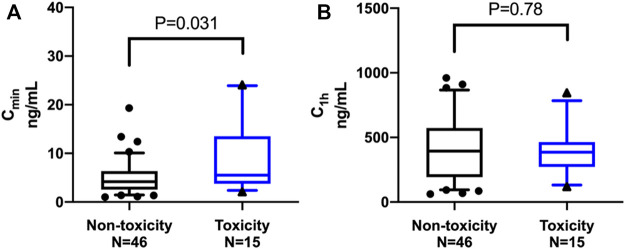
Comparison of lenalidomide plasma concentrations between the groups. The middle line represents the median in each group. **(A)** The trough concentration (C_min_), expressed as the median (IQR), was significantly higher in the toxicity group than in the non-toxicity group [5.53 (3.97–13.05) ng/mL versus 4.17 (1.03–6.33) ng/mL; *p =* 0.031]. **(B)** The plasma concentration at 1 h (C_1h_) after oral administration, expressed as the mean (SD), showed no significant difference between the toxicity group and the non-toxicity group [396.67 (206.73) ng/mL versus 416.98 (254.05) ng/mL; *p =* 0.78].

Figure 2 shows the comparison of LEN plasma concentrations between the groups. The trough concentration of LEN at 0.5 h before oral administration (C_min_), expressed as the median (IQR), was significantly higher in the toxicity group [5.53 (3.97–13.05) ng/mL] than in the no-toxicity group [4.17 (1.03–6.33) ng/mL; *p* = 0.031]. Regarding the plasma concentration of LEN at 1 h after oral administration (C_1h_), expressed as the mean (SD), there was no significant difference between the toxicity group [396.67 (206.73) ng/mL] and the no-toxicity group [416.98 (254.05) ng/mL; *p* = 0.78].

### ROC Curve for Hematological Toxicity

**FIGURE 3:**
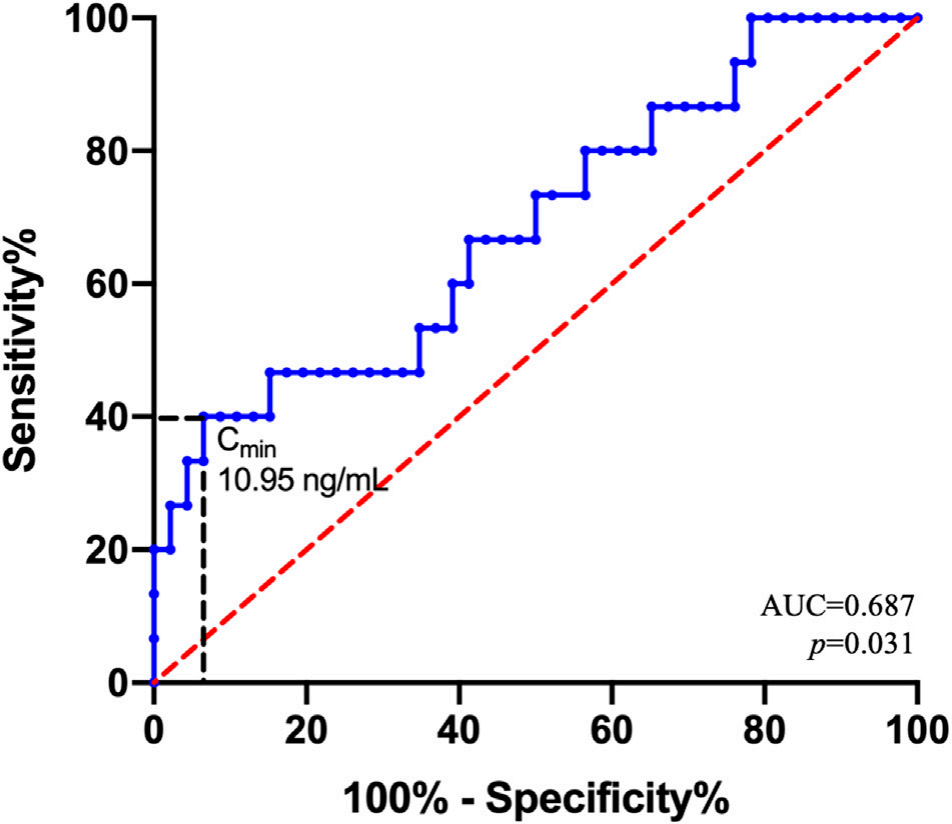
Receiver operating characteristic (ROC) curve for hematological toxicity. ROC curve estimates for the 61 patients. AUC is the area under the ROC curve. With regard to the C_min_ threshold value of 10.95 ng/mL, the sensitivity and specificity were 0.4 and 0.935, respectively.

Using the ROC curve (Figure 3), the LEN C_min_ threshold predicting hematological toxicity was 10.95 ng/mL with an area under the curve (AUC) of 0.687 [95% confidence interval (95% CI) = 0.527–0.847; *p* = 0.031]. Considering the threshold value of 10.95 ng/mL, the sensitivity and specificity were 0.4 and 0.935, respectively.

Then, the LEN C_min_ was binarized according to the 10.95 ng/mL threshold as “high exposure” when C_min_ was above this value and as “low exposure” when below. Compared to the “low exposure” group (*n* = 9/52, 17.31%), there was a significantly increased risk of toxicity in the “high exposure” group (*n* = 6/9, 66.67%; *p* = 0.006).

### Factors Associated With Hematological Toxicity

Logistic regression analysis was used to identify independent factors influencing hematological toxicity. The results of the univariate and multivariate analyses are presented in Table 2. The dichotomized LEN C_min_ was retained in the final model. In line with previous analysis, a LEN C_min_ threshold below 10.95 ng/mL was significantly associated with a decreased risk of hematological toxicity (<10.95 ng/mL vs. >10.95 ng/mL: OR = 0.023, 95% CI = 0.002–0.269; *p* = 0.003). In other words, compared to “low exposure” (C_min_<10.95 ng/mL), “high exposure” (C_min_>10.95 ng/mL) was associated with an apparent increase in the odds of developing hematological toxicity.

**TABLE 2:** Univariate and multivariate analyses of factors influencing hematological toxicity.

Characteristics	Univariate analysis		Multivariate analysis	
	OR (95% CI)	*p* value	OR (95% CI)	*p* value
Male (vs. female)	0.545 (0.161–1.847)	0.330	0.255 (0.030–2.185)	0.212
Age (years)	0.976 (0.925–1.030)	0.374	0.998 (0.911–1.093)	0.965
Weight (kg)	1.025 (0.971–1.082)	0.374	0.991 (0.898–1.092)	0.850
BMI (kg/m^2^)	1.098 (0.917–1.314)	0.310		
BSA (m^2^)	3.393 (0.103–112.125)	0.494		
MM (vs. NHL)	0.157 (0.044–0.559)	0.004*	0.342 (0.057–2.046)	0.240
SCr (μmol/L)	1.004 (0.972–1.037)	0.807		
CCr (mL/min)	1.007 (0.990–1.025)	0.428	1.012 (0.973–1.053)	0.541
Total protein (g/L)	1.004 (0.958–1.052)	0.873		
Albumin (g/L)	1.131 (0.981–1.304)	0.090	1.182 (0.918–1.522)	0.195
ALT (U/L)	1.010 (0.977–1.043)	0.558		
AST (U/L)	0.976 (0.915–1.041)	0.466		
ALP (U/L)	0.978 (0.950–1.007)	0.142		
Courses of Lenalidomide (n)	0.966 (0.785–1.188)	0.742		
Lenalidomide duration (days)	0.965 (0.828–1.126)	0.655		
Lenalidomide dosage				
25 mg QD	References			
Less than 25 mg QOD	0.923 (0.249–3.428)	0.905		
Co-administration of targeted therapy^a^ (vs. no)	0.244 (0.059–1.008)	0.051		
Co-administration of glucocorticoids (vs. no)	0.317 (0.093–1.089)	0.068		
Co-administration of cytotoxic antitumor drugs (vs. no)	5.250 (1.190–23.171)	0.029*	8.331 (0.905–76.703)	0.061
Co-administration of aspirin (vs. no)	1.786 (0.546–5.839)	0.337		
Co-administration of antiviral agents (vs. no)	0.517 (0.143–1.870)	0.314		
Co-administration of antibacterial agents (vs. no)	2.026 (0.500–8.207)	0.323		
Co-administration of PPI or H2RA (vs. no)	4.100 (0.992–16.950)	0.051		
C_min_ (<10.95 vs. >10.95 ng/mL)	0.143 (0.029–0.700)	0.016*	0.023 (0.002–0.269)	0.003*
C_1h_ (ng/mL)	1.000 (0.997–1.002)	0.776		

Abbreviations; CI, Confidence interval; BMI, body mass index; BSA, body surface area; MM, Multiple myeloma; NHL, Non-Hodgkin lymphoma; SCr, Serum creatinine; CCr, Creatinine clearance; ALT, Alanine aminotransferase; AST, Aspartate aminotransferase; ALP, Alkaline phosphatase; QD, Once a day; QOD, Every other day; PPI, Proton pump inhibitor; H2RA, H2 Receptor Antagonist.

a Targeted therapy includes bortezomib, isazomi, ibrutinib, zanubrutinib, orelabrutinib, rituximab and obinutuzumab.

## Discussion

### General Findings and Trends

LEN, a non-chemotherapy immunomodulator, has been extensively used in the treatment of MM and NHL, and the mechanism involves direct cytotoxicity as well as indirect effects on tumor immunity (Gribben et al., 2015). With the expanding role of LEN in hematologic malignancies, the management of its hematological toxicity has become a wide clinical concern and research focus (Cheson et al., 2020). Despite dose adjustments according to the severity of myelosuppression, unacceptable hematological toxicity is still the most common factor preventing continuous therapy with LEN. To date, there is no established and feasible marker that can be used as a predictive factor in routine clinical practice to inform a high risk of developing hematological toxicity. Therefore, we paid more attention to the association between hematological toxicity and its plasma concentration in this study.

This current study revealed that only C_min_ was independently associated with hematological toxicity. We found that LEN over-exposure contributed to its hematological toxicity, which is similar to previous investigations on the cumulative exposure (AUC) of LEN (Komrokji et al., 2012; Chen et al., 2013; Kobayashi et al., 2018). Given that a C_min_ higher than 10.95 ng/mL was associated with a remarkable increase in the risk of developing toxicity, a C_min_ of 10.95 ng/mL was determined as the upper limit to prevent hematological toxicity. This threshold was associated with a specificity of 93.5% and a sensitivity of 40%.

Conversely, no apparent association was observed between LEN C_1h_ and its hematological toxicity in our study, which corresponds to the findings in a previous investigation on the relationship of peak serum concentration (C_max_) and hematological toxicity (Bridoux et al., 2016). However, it is notable that the inter-individual variability of the time to reach C_max_ (T_max_) could not be excluded; thus, C_1h_ cannot be equal to C_max_ in our study.

As similar pharmacokinetic profiles of LEN have been previously shown in patients with various types of hematological malignancies (Chen et al., 2017), the two most common hematologic malignancies, MM and NHL, were simultaneously included in our study population. Interestingly, the results of univariate analysis suggested that patients with MM might have a lower risk of hematological toxicity than those with NHL, which needs to be studied further. In addition, the univariate analysis suggested a possible tendency toward an increased risk of hematological toxicity in patients co-administered cytotoxic antitumor drugs. However, it was not an independent factor affecting hematological toxicity in the multivariate analysis, which also enhances the reliability of the observed association between C_min_ and hematological toxicity.

### Key Strengths and Significance

With regard to TDM, sampling trough concentrations at steady state (C_min_) is often performed in clinical practice and is currently the most precise approach, as it avoids the shrinkage of individual information to the population mean (Mueller- Schoell et al., 2021). To the best of our knowledge, the present study is the first to establish and highlight a relationship between LEN C_min_ and its hematological toxicity. Furthermore, multivariate logistic analysis confirmed the significance of this cut-off value of LEN C_min_, and we propose it as an optimal index for TDM of LEN hematological toxicity. In comparison, TDM based on cumulated AUC requires collecting and analyzing multiple blood samples, whereas dense blood sampling is rarely feasible in the clinical setting.

Of note, despite TDM’s value in oncology becoming more recognized, it is still not commonly used in antineoplastic treatment compared to other therapeutic areas (e.g., antimicrobial and antiepileptic) (Wicha et al., 2015; Velghe and Stove, 2018; Mueller- Schoell et al., 2021). The present study provides exploratory evidence for LEN TDM in patients with NHL and MM, which contributes to further promoting the extensive use of TDM in the field of oncology. Additionally, the exposure-safety relationship was established based on real-world data from patients’ medical records in our study. Evidence from the real-world setting can help to establish a broad picture of TDM implementation in everyday clinical practice.

### Recommendations for Clinical Practice

From a clinician’s or pharmacist’s point of view, the hematological toxicity of LEN has become a wide clinical concern. Herein, we discuss recommendations regarding the clinical management of the hematological toxicity of LEN. First, prior to the initiation of LEN treatment, the assessment of patients’ baseline kidney function, complete blood count (CBC), and other concomitant drugs causing myelosuppression (e.g., cytotoxic drugs) should be performed. These aforementioned factors should be taken into consideration when determining the initial dose. Second, early measurement of LEN C_min_ on at least day 3 after starting LEN therapy (that is, at least 2 days of dosing) would help to inform a high risk of developing hematological toxicity. Then, individual dose adjustments can be made if necessary. Last but not least, patients should be well trained and motivated to take their medication correctly. Additionally, patients should have CBC assessment regularly to monitor for hematological toxicity, particularly neutropenia.

### Limitations and Future Perspectives

Several limitations should be considered for our study. First, we included only a relatively small number of patients; thus, the findings need to be confirmed in a larger and independent population. The outcome measure of hematology toxicity was defined as grade 2 and a higher level in this study, whereas the relationship between exposure and severe hematology toxicity (grade 3 and above) still deserves further consideration. Second, it was unfeasible to assess the exposure-response relationship due to lack of follow-up on long-term efficacy. More particularly, efficacy is a multifactorial and more complex process than toxicity events. Future well-designed studies are warranted to explore the exposure-response relationship. Third, patients were required to delay the breakfast on the morning of the blood sample, but it was difficult to be sure that these instructions were followed. However, it is unlikely that this would alter our conclusions of C_min_.

## Conclusion

In conclusion, we demonstrate that the LEN trough concentration correlates with hematological toxicity, and the C_min_ threshold for hematological toxicity (10.95 ng/mL) is proposed. These findings provide the first elements of proof in favor of C_min_-based TDM in NHL or MM patients receiving LEN therapy. Altogether, LEN TDM appears to be a new approach to improve medication safety and achieve continuous treatment for NHL or MM patients in routine clinical care.

## Data Availability

The original contributions presented in the study are included in the article/supplementary material, further inquiries can be directed to the corresponding authors.
